# The Costs of Online Learning: Examining Differences in Motivation and Academic Outcomes in Online and Face-to-Face Community College Developmental Mathematics Courses

**DOI:** 10.3389/fpsyg.2019.02054

**Published:** 2019-09-10

**Authors:** Michelle K. Francis, Stephanie V. Wormington, Chris Hulleman

**Affiliations:** Curry School of Education and Human Development, University of Virginia, Charlottesville, VA, United States

**Keywords:** developmental mathematics, community college, online learning, academic motivation, adult learners

## Abstract

Although online courses are becoming increasingly popular in higher education, evidence is inconclusive regarding whether online students are likely to be as academically successful and motivated as students in face-to-face courses. In this study, we documented online and face-to-face students’ academic motivation and outcomes in community college mathematics courses, and whether differences might vary based on student characteristics (i.e., gender, underrepresented ethnic/racial minority status, first-generation college status, and adult learner status). Over 2,400 developmental mathematics students reported on their math motivation at the beginning (Week 1) and middle (Weeks 3, 5) of the semester. Findings indicated that online students received lower grades and were less likely to pass from their courses than face-to-face students, with online adult learners receiving particularly low final course grades and pass rates. In contrast, online and face-to-face students did not differ on incoming motivation, with subgroup analyses suggesting largely similar patterns of motivation across student groups. Together, findings suggest that online and face-to-face students may differ overall in academic outcomes but not in their motivation or differentially based on student characteristics. Small but significant differences on academic outcomes across modalities (Cohen’s *d*s = 0.17–0.28) have implications for community college students’ success in online learning environments, particularly for adult learners who are most likely to be faced with competing demands.

## Introduction

Online courses are increasingly popular in higher education, with over 3 million students nationwide having participated in at least one online course (Bennett and Monds, 2008; Green and Wagner, 2011). Growing access to online learning holds promise for Science, Technology, Engineering, and Mathematics (STEM) education, as online math and science courses augment both the number and diversity of students entering into STEM majors (Drew et al., 2015). Online courses also offer increased flexibility for non-traditional learners, such as adult learners, in terms of scheduling and transportation (Hung et al., 2010; Yoo and Huang, 2013). However, there is contradictory evidence on whether students fare equally well in online courses as they do in face-to-face courses. Some studies indicate no differences in performance by modality (e.g., online versus face-to-face courses, Bernard et al., 2004; O’Dwyer et al., 2007; Driscoll et al., 2012). Other studies, by contrast, suggest that online students drop out more often than their face-to-face counterparts (Patterson and McFadden, 2009; Boston and Ice, 2011). Low achieving students may face particular difficulties in online courses (Harrington, 1999), raising the question of whether online courses are benefitting the students who are most likely to enroll in them. Given the increasing popularity and contradictory evidence associated with online learning, it is critical to understand who is likely to be successful in online courses and the underlying mechanisms that may explain differential success.

Students’ academic motivation, or reasons for engaging in a task, is an important predictor of academic success that has been under-investigated in the online learning space (Jones and Issroff, 2005; Ortiz-Rodríguez et al., 2005; Yoo and Huang, 2013). In the current study, we examined whether community college students enrolled in online and face-to-face math courses received different academic outcomes, and whether any differences might be a function of students’ incoming motivation and changes in motivation. We focused on developmental mathematics courses, which are designed for students who place below college-level math, because they are characterized by notoriously low pass rates and serve as a significant barrier to degree completion (Bailey et al., 2010; Hughes and Scott-Clayton, 2011).

### Learning Context: Community College Developmental Mathematics Courses

We chose to conduct this study in developmental mathematics courses at a community college given the high-stakes nature of developmental mathematics courses, dearth of evidence on online learning in community college settings and exponential growth of online courses in community colleges (Johnson and Mejia, 2014; Allen and Seaman, 2016). Community colleges serve more than one-third of degree-seeking U.S. citizens, and include proportionally more students from underrepresented groups (Levin, 2007; Ma and Baum, 2016). Over half of community college students are placed into at least one developmental course, which are designed for students who place below the college-ready level. Developmental math courses serve as a prerequisite to credit-bearing mathematics classes, are characterized by notoriously low pass rates, and can serve as a barrier to degree completion (Bailey et al., 2010; Hughes and Scott-Clayton, 2011). For instance, Silva and White (2013) reported that over 75% of students in developmental courses fail to achieve college-level readiness even after several semesters of remediation. As such, community college developmental mathematics courses serve as a high-stakes area to understand the effect of online learning on academic motivation and outcomes.

### Online Learning Environments

Over the past two decades, online learning enrollment has consistently outpaced traditional student enrollment (Oblinger and Hawkins, 2005; Allen and Seaman, 2016), with the number of students enrolled in at least one online course increasing by 32% from 2006 to 2008 (Bennett et al., 2010). These trends are most pronounced in community colleges, higher education institutions which offer 2 years associate’s degrees (Allen and Seaman, 2016). Community colleges accounted for more than one-half of all online enrollments from 2002 to 2007, with one in five students at such institutions taking at least one online course between 2011 and 2012 (Johnson and Mejia, 2014). Given the impressive growth in online course enrollment, it is necessary to gain a better understanding of whether online learning is beneficial or detrimental to student success. Some researchers suggest that students fare better when engaging in online courses. For instance, Feintuch (2010) concluded from a review of more than 1000 articles that students in online learning environments spent more time engaged with course materials. She also argued that online students could benefit from personalized instruction and real-time feedback. Similarly, other researchers have lauded the potential for online course offerings to support enrollment among traditionally underserved students (e.g., adult learners, underrepresented ethnic/racial minority students) due to increased flexibility (Hung et al., 2010; Yoo and Huang, 2013), particularly in fields with a lack of diversity such as STEM (Drew et al., 2015). By contrast, other researchers have argued that students enrolled in online courses perform worse than they would in face-to-face courses. For example, researchers cite that dropout rates can be as much as 10–20% higher in online courses than in comparable face-to-face courses (Harris and Parrish, 2006; Xu and Jaggars, 2013). Still other researchers report no difference between academic outcomes in face-to-face and online learning environments (e.g., Bernard et al., 2004; Steinweg et al., 2005; Zhao et al., 2005).

To complicate matters further, researchers have called into question the academic rigor of existing studies. For example, Phipps and Merisotis (1999) noted that many studies failed to use valid and reliable measures or account for students’ attitudes. Other scholars assert that conclusions depend on how academic success is operationalized. For example, findings from the Public Policy Institute of California indicated that online learning was correlated with negative short-term outcomes, including course pass rates that were 11–14% lower than face-to-face courses even when controlling for overall grade point average and school characteristics. However, the same report suggested that participating in an online course may have long-term benefits for community college students, including greater likelihood to attain an associate’s degree and enroll in a 4 years institution (Johnson and Mejia, 2014).

#### Differences by Student Characteristics

Researchers are increasingly attending to the fact that online learning may be more beneficial for some types students compared to others. One group that has received substantial attention is adult learners (i.e., individuals who are 24 or older; Yoo and Huang, 2013). On the one hand, online courses provide increased access for adult learners, who are more likely to have work and family obligations to balance alongside attaining a degree (Hung et al., 2010; Yoo and Huang, 2013). Adult learners may be expected to perform better in online courses because they tend to be more self-directed and autonomous (Arghode et al., 2017). On the other hand, they may be less familiar navigating online learning environments, which can affect their performance (Chang, 2015; Lai, 2018).

Results comparing success rates for adult and traditional-aged learners are inconsistent. A number of studies found that adult learners perform more poorly in online learning environments than in face-to-face learning environments (Richardson and Newby, 2006; Park and Choi, 2009; Yoo and Huang, 2013). For example, one study found that online adult learners enrolled in an MBA program were four times more likely to drop out than in face-to-face courses (Patterson and McFadden, 2009). Other studies have found that adult learners participate more often in course activities than traditional-aged learners (Kilgore and Rice, 2003; Hoskins and Van Hooff, 2005; Chyung, 2007) or found that age was not a significant predictor of outcomes (Hargis, 2001; Ke and Xie, 2009). Taken together, findings related to age and success in online courses are inconsistent. The current study not only considered academic outcomes for adult learners compared to traditional-aged students, but also characterized their incoming motivation levels.

Other student characteristics that have been examined with respect to online learning are gender (e.g., Park, 2007; Cercone, 2008) and prior achievement (e.g., Harrington, 1999; Summers et al., 2005). With respect to gender, findings suggest that females may be more actively engaged in online learning (e.g., Chyung, 2007), but this does not translate to lower dropout rates (Park and Choi, 2009). With respect to prior achievement, findings suggest that lower achieving students may be less satisfied with online compared to face-to-face courses (e.g., Summers et al., 2005) and may perform more poorly in online than in face-to-face courses or than their higher achieving classmates. Relevant to the current study, which was conducted in developmental mathematics courses, Harrington (1999) examined students’ performance in online and face-to-face versions of an introductory statistics course. She found that students with low grade point averages performed more poorly in the online course than low-performing students in the face-to-face course, or than high-achieving students in either version of the course. Overall, evidence suggests some potential differences in how successful students may be in online courses based on their individual characteristics. However, the evidence is generally mixed and several important student characteristics have yet to be systematically examined (e.g., underrepresented ethnic/racial minority status, first generation college status).

### Academic Motivation in Online Learning Contexts

The research on non-cognitive factors in online education has focused heavily on students’ behaviors, such as self-regulated learning strategies, as predictors of success in online learning environments (Broadbent and Poon, 2015). However, differences between online learning and face-to-face environments may also affect students’ attitudes in online courses (Rovai, 2002; Baker, 2004; Mullen and Tallent-Runnels, 2006). Online students may require adaptive motivation to stay engaged (Karimi, 2016; Park and Yun, 2018) and enrolled in their courses (Aragon and Johnson, 2008) more so than face-to-face students, and online courses may attract students with lower initial motivation. This study documents online learners’ motivation in comparison to face-to-face students in the same courses.

To operationalize motivation, we adopted an expectancy-value-cost framework (Eccles, 1983; Barron and Hulleman, 2015) because it is well established, describes student motivation broadly, and aligns with constructs from popular conceptualizations of motivation in the online learning space (e.g., Keller and Suzuki, 2004). Expectancy-value-cost theory posits that the most proximal determinants of student motivation are expectancy (i.e., belief that one can complete a task successfully), value (i.e., belief that there is a worthwhile reason for engaging in a task), and cost (i.e., belief that that there are obstacles preventing one from engaging in a task). Because we were interested in capturing a rich description of students’ motivation, we assessed a number of related motivational constructs (for a similar approach, see Hulleman et al., 2016). Related to expectancy, we assessed growth mindset (i.e., belief that intelligence is malleable and can be improved; Dweck and Leggett, 1988; Dweck, 2006). Related to value, we assessed interest (i.e., engaging in a task due to interest or enjoyment; Eccles, 1983) and social belonging (i.e., belief that one fits in to the learning context and is respected by others in that environment; Cohen and Garcia, 2008).

A growing number of studies have assessed online learners’ expectancy or value. Consistent with the broader literature, expectancy-related constructs – particularly self-efficacy, or students’ perception that they can successfully complete a task – were positively associated with online students’ course satisfaction (Brinkerhoff and Koroghlanian, 2007), performance (Joo et al., 2000; Wang and Newlin, 2002; Lynch and Dembo, 2004; Bell and Akroyd, 2006), persistence (Holder, 2007), and likelihood of enrolling in future online courses (Lim, 2001; Artino, 2007). Overall perceived value of course content was also associated with course satisfaction (Xie et al., 2006), performance (Yoo and Huang, 2013), and future enrollment choices (Artino, 2007), although some articles reported no associations between value and final grade (Chen and Jang, 2010). Studies suggested that value was a particularly important predictor for adult learners (Kim and Frick, 2011). There is less empirical evidence suggesting that perceived cost is associated with poorer academic outcomes. However, theory suggests that cost may be lower in online courses since they do not require students to be in a particular location at a particular time. Cost may be a critical predictor for certain groups of online learners, such as adult learners who may be more likely to be balancing competing demands on their time from work, family, and school (Hung et al., 2010).

### Current Study

We sought to document academic outcomes, incoming motivation, and changes in motivation for students enrolled in online and face-to-face math courses. We were interested in two primary research questions. First, do students enrolled in online courses receive lower academic outcomes (i.e., final grades, pass rates, withdraw rates) than students enrolled in face-to-face courses? We hypothesized that online students would receive lower final grades and pass rates, but higher withdraw rates, than face-to-face students (Patterson and McFadden, 2009) based on evidence that lower achieving students struggle in online learning environments (Harrington, 1999; Coldwell et al., 2008) and community college students reported negative short-term outcomes in online courses (Johnson and Mejia, 2014).

Second, do online students report lower incoming motivation than face-to-face students, and does that vary Eccles, 1983 as a function of student characteristics (i.e., gender, adult learner status, underrepresented ethnic/racial minority status, and generation status)? Given the general lack of evidence in online courses in general, and community college developmental math courses in particular, we tentatively hypothesized that online students would report (1) lower perceived cost than face-to-face students, given the argument that online courses offer increased flexibility Yoo and Huang, 2013); and (2) lower belonging, given that online courses tend to involve less interaction and synchronous learning opportunities.

## Materials and Methods

### Participants

The sample included 2,411 students (*M*_age_ = 20.7 years, *SD*_age_ = 5.2 years) from a community college in the Southeastern United States. Participants were enrolled in 310 individual courses of two different developmental mathematics topics – Intermediate Algebra and College Math – taught by 63 instructors over six semesters. Participants were drawn from the control condition of a larger randomized-control trial assessing the effects of a utility-value and growth mindset intervention on students’ math achievement. Participants were primarily female (60%), with 70% having applied for financial aid, 50% identifying as first-generation students (i.e., neither parent received a degree form a 4 years institution), 45% identifying as part-time students, and 13% adult learners. Approximately 31% self-reported as White, 38% Hispanic/Latino, 21% Black/African American, 2.1% Asian, and 7.9% reporting another ethnicity. The current sample is representative of the overall population of the community college (31% White, 32% Hispanic/Latino, 18% Black/African American, 6% Asian, and 13% reporting some other ethnicity). Out of the total sample, 2,036 students (84.45%) were enrolled in face-to-face courses and 375 (15.55%) were enrolled in online courses. Students in online courses were more likely to be women (66%), identify as an underrepresented ethnic/racial minority group (53%; i.e., identifying as Hispanic/Latino, Black/African American, or Pacific Islander at this institution), adult learners (i.e., 25 or older; 26%) and enrolled part time (52%) than students in face-to-face courses [59% female, χ^2^(1, *N* = 2,384) = 6.18, *p* = 0.013; 43% underrepresented ethnic/racial minority, χ^2^(1, *N* = 2,227) = 17.17, *p* < 0.001; 11% adult learner, χ^2^(1, *N* = 2,411) = 0.65.41, *p* < 0.001; 43% part time learner, χ^2^(1, *N* = 2,411) = 9.52, *p* = 0.002]. Students in online and face-to-face courses did not differ by generation status.

### Measures

#### Academic Motivation

Student motivation was assessed via self-report survey measures for four constructs from Expectancy-Value-Cost Theory – expectancy, value, cost, and interest – at four points throughout the semester, along with growth mindset and social belonging at the beginning of the semester. Four expectancy items (α = 0.90; e.g., “How confident are you that you can learn the material in the class?”), six value items (α = 0.91; e.g., “How important is this class to you?”), five cost items (α = 0.78; e.g., “How stressed out are you by your math class?”), and three interest items (α = 0.88; e.g., “How interested are you in learning more about math?”) were adapted from the Expectancy-Value-Cost Scale (Kosovich et al., 2015; Hulleman et al., 2017) to make them specific to math courses. Responses ranged from 1 (*Not at All*) to 6 (*Extremely*). Students also completed a three-item measure of growth mindset from Good et al. (2012), (α = 0.85; e.g., “I have a certain amount of math ability, and I can’t really do much to change it”; reverse-scored) on a 6-point Likert-type scale (1 = *Strongly Disagree*; 6 = *Strongly Agree*) and a three-item measure of social belonging (Manai et al., 2016; α = 0.75; e.g., “In this class, how much do you feel as though you belong?”) on a 6-point Likert-type scale (1 = *Not at All*; 6 = *Extremely*). Confirmatory factor analysis indicated that the motivation variables fit the data well (χ^2^(231, *N* = 1,676) = 1220.54, *p* < 0.001; CFI: 0.96; TLI: 0.95; RMSEA: [0.047, 0.053]; SRMR: 0.043).

#### Academic Outcomes

Administrative data were collected from the office of institutional research at the end of the semester for pass rates, withdraw rates, and numeric grade. Pass rates were calculated such that students who earned an A, B, or C in the course were coded a “1” while students who earned a D, F, W, or I were coded as “0.” Withdraw rates were calculated such that students who earned a Withdraw (W) in the course were coded as a “1” while students who earned an A, B, C, D, or F were coded as a “0.” Students who earned an incomplete (I, *n* = 4 students) could not be categorized. Numeric grades were coded by converting letter grades to a normal GPA scale (0–4) such that students who received an A were coded as a “4,” students who received a B were coded as a “3,” students who received a C were coded as a “2,” students who received a D were coded as a “1,” students who received an F or a W were coded as a “0.”

### Procedure

All students enrolled in participating developmental mathematics courses were invited to participate in the current study. Materials were administered online through the Qualtrics platform during the lab portion of students’ developmental mathematics class for the face-to-face courses, and as part of an assigned homework activity on the course management platform for online courses. Overall, 91% of students enrolled in participating courses completed at least one of the 4 activities. Participants reported on their motivation during the first class period of the semester in Week 1 (Time 1, 70% response rate), as well as during Week 3 (Time 2, 77% response rate), Week 5 (Time 3, 70% response rate), and Week 12 (Time 4, 45% response rate). Given the low response rate, we did not consider motivation at Time 4 in analyses for the current study. After responding to survey items, participants provided information about their self-identified gender, race, parental education, and previous academic achievement before being thanked for their time. Instructors incentivized students to complete activities with course credit, but were given autonomy over what kind of course credit they offered (e.g., extra credit, participation grade).

### Analytic Plan

Descriptive differences in student achievement, demographics, and baseline motivations by course modality were examined by conducting *t*-tests and ANOVAs Because modality comparisons were exploratory, we employed a Bonferroni adjustment for them and reduced our threshold for significance to α = 0.0056 (see Table 1). Additionally, we calculated effect sizes of differences (i.e., Cohen’s *d*) to consider practical significance. See the Supplementary Materials for the tables displaying descriptive differences in student achievement, demographics, and baseline motivation by course modality and student characteristics (gender, underrepresented ethnic/racial minority status, generation status, and adult learner status).

**TABLE 1:** Variables of interest by course modality.

	**Face-to-**			**Effect size**
	**face**	**Online**	***T*-test**	**(Cohen’s d)**

	**Mean (*SD*)**			
**Academic Outcomes**				
Pass rate	0.66 (0.47)	0.54 (0.50)	*t*(506) = −4.23, *p* < 0.001	*d* = −0.25
Grade	2.06 (1.46)	1.65 (1.52)	*t*(508) = −4.91, *p* < 0.001	*d* = −0.28
Withdraw	0.13 (0.33)	0.18 (0.39)	*t*(479) = 2.69, *p* = 0.007	*d* = 0.17
rate				
**Baseline Motivation**				
Expectancy	3.81 (0.81)	3.66 (0.85)	*t*(360) = −2.65, *p* = 0.008	*d* = −0.18
Value	3.58 (0.92)	3.59 (0.93)	*t*(369) = 0.13, *p* = 0.894	*d* = 0.01
Cost	2.50 (0.84)	2.66 (0.85)	*t*(368) = 2.75, *p* = 0.006	*d* = 0.19
Relevance	3.20 (1.16)	3.13 (1.19)	*t*(366) = −0.82, *p* = 0.414	*d* = −0.06
Interest	2.71 (1.19)	2.64 (1.20)	*t*(368) = −0.89, *p* = 0.372	*d* = −0.06
Growth	3.88 (1.20)	3.81 (1.30)	*t*(354) = −0.75, *p* = 0.454	*d* = −0.05
mindset				
Belonging	3.67 (0.77)	3.58 (0.80)	*t*(363) = −1.63, *p* = 0.104	*d* = −0.11

To determine whether students in online and face-to-face courses received different academic outcomes, we tested the influence of course modality, the interaction of course modality and student characteristics, and the latent interaction of baseline motivation and course modality on student academic achievement in the course. To determine whether students in online courses were less motivated initially than students in face-to-face courses, we tested the influence of course modality and the interaction of course modality and student characteristics on latent student baseline motivation. To do this, we fit two structural equation models – one predicting the three academic outcomes (i.e., pass rates, withdraw rates, and numeric grade) and one predicting the six latent student motivation scores (i.e., expectancy, value, cost, interest, growth mindset, and belonging). Models included course modality, course modality and student demographic interactions, and course modality and latent incoming motivation interactions (only when predicting academic achievement) as predictors. Models were estimated in the statistical program R using the “lavaan” package (Rosseel, 2012).

For all analyses predicting latent baseline student motivation, we controlled for student gender (i.e., male versus female), student underrepresented minority status (i.e., Hispanic/Latino or Black/African American versus White or Asian), student generation status (i.e., first-generation status versus continuing-generation status, adult learner status, and prior achievement (i.e., high school GPA). For all analyses predicting academic outcomes (i.e., pass rate, numeric grade, withdraw rate), we controlled the same student covariates as well as the six latent student motivation scores.

To determine if students in face-to-face courses or online courses experienced differences in their change in motivation over the course of the semester, we tested the influence of course modality on Time 3 student motivation while accounting for Time 1 student motivation. These models could not be estimated in an SEM framework, as the sample size of online students participating during surveys conducted during both Time 1 and Time 3 was too small. To answer this question, models were estimated in the statistical program R using the “lme4” package (Bates et al., 2014). This package is appropriate for cross-classified levels in data structures, which was necessary for the current study given that instructors taught courses across multiple semesters. Prior to analyses, all continuous predictor variables (e.g., Time 1 motivation composites, student’s reported high school GPA) were grand-mean centered. For all analyses predicting changes in motivation over the semester (i.e., Time 3 expectancy, value, cost, and interest), we controlled for the aforementioned student covariates along with students’ Time 1 composite score for the motivational construct being predicted.

## Results

### Predicting Academic Outcomes by Course Modality

**FIGURE 1:**
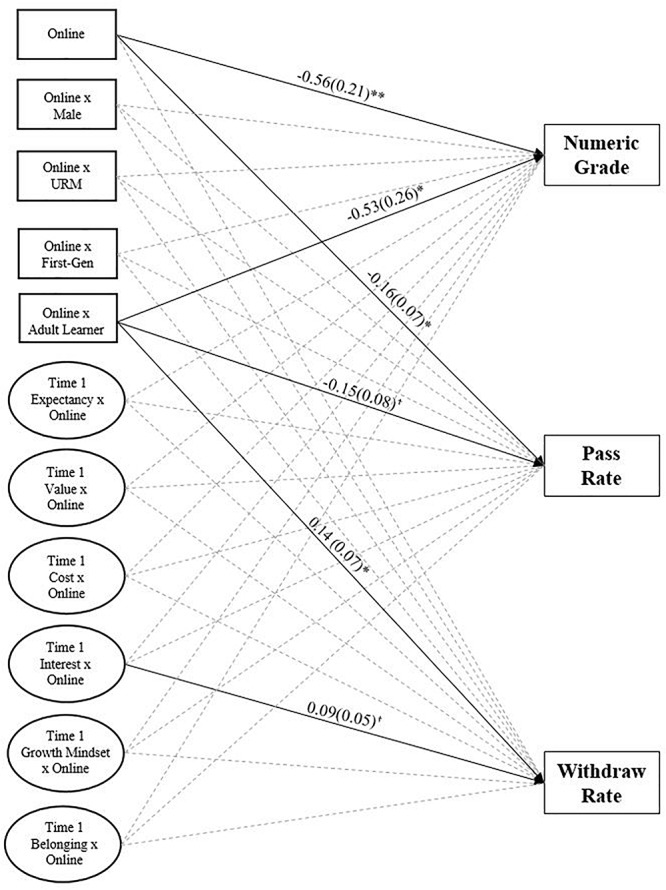
Course modality and interactions predicting academic outcomes. ^†^*p* < 0.10, ^∗^*p* < 0.05, and ^∗∗^*p* < 0.01.

First, we tested whether course modality predicted students’ course performance (i.e., whether students in online courses performed better, worse, or the same as students in face-to-face courses). Descriptive statistics for course performance by course modality can be seen in Table 1. Structural equation models were conducted in which pass rate and withdraw rate were predicted by course modality (0 = face-to-face; 1 = online). All models controlled for latent baseline student motivation scores, student gender, student underrepresented ethnic/racial minority status, student generation status, and student prior achievement. The model fit the data well (χ^2^(1,480, *N* = 1,456) = 5048.12, *p* < 0.001; CFI: 0.92; TLI: 0.91; RMSEA: [0.039, 0.042]; SRMR: 0.041). As shown in Figure 1, being enrolled in an online course was significantly negatively associated with pass rate and numeric grade (β = −0.56, *p* = 0.007; β = −0.16, *p* = 0.021) but did not predict withdraw rate.

This same model also included interactions of course modality and student characteristics to determine if the effect of course modality on academic achievement was differential by gender, student underrepresented racial/ethnic minority status, generation status, or adult learner status. As shown in Figure 1, being an adult learner in an online course was significantly negatively associated with numeric grade (β = −0.53, *p* = 0.043), marginally negatively associated with pass rate (β = −0.15, *p* = 0.064), and significantly predicted withdraw rate (β = 0.14, *p* = 0.043). These results suggest that students in online courses tend to pass less often, withdraw more often, and earn lower numeric grades than students in face-to-face courses. Further, this effect is not a function of student gender, underrepresented ethnic/racial minority status, or generation status but may be a function of adult learner status.

### Modality Effects on Academic Outcomes Based on Incoming Motivation

One hypothesis was that differences in online and face-to-face students’ performance is a function of their incoming motivation. To assess this possibility, we included latent interactions of baseline motivation and course modality for each motivation construct (i.e., expectancy, value, cost, interest, growth mindset, and belonging) predicting academic achievement. As shown in Figure 1, there were no significant interactions of course modality and Time 1 motivation predicting academic outcomes with the exception that being an online student scoring higher on interest in the course was marginally associated with withdraw rate (β = 0.09, *p* = 0.080). These results suggest that the differences in online and face-to-face students’ academic performance is not a function of their baseline motivation coming into the course with the marginal exception of perceived interest in the course when considering withdraw rate.

### Baseline Motivational Differences by Course Modality

Next, we examined whether students’ incoming motivation significantly differed based on course modality (i.e., whether students in online courses were more, less, or equally motivated at the beginning of the semester as students in face-to-face courses). As displayed in Table 1, students in online courses did not differ significantly from students in face-to-face courses in any of the Time 1 motivational constructs. In terms of practical significance, effect sizes indicated that any differences were below what would be considered a small effect (i.e., Cohen’s *d*s < 0.30). Results suggested that face-to-face and online students did not differ in their incoming motivation. This was further supported by the SEM model predicting latent incoming student motivation scores, which fit the data well (χ^2^(414, *N* = 1,456) = 1393.28, *p* < 0.001; CFI: 0.95; TLI: 0.94; RMSEA: [0.038, 0.043]; SRMR: 0.033). with the exception of online course enrollment being marginally negatively associated with latent incoming interest scores (β = −0.28, *p* = 0.098), there were no differences between online and face-to-face students in academic motivation.

**FIGURE 2:**
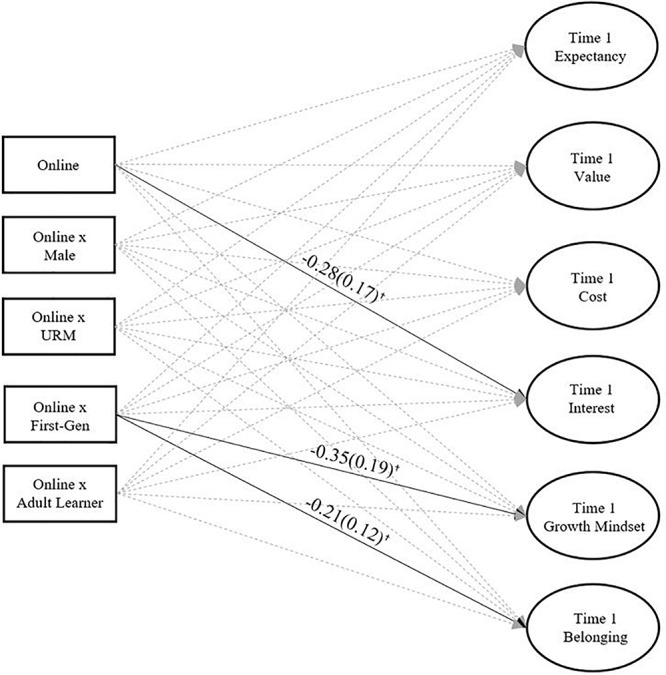
Course modality and interactions of course modality and student characteristics predicting latent baseline student motivation. ^†^*p* < 0.10.

We were also interested in determining whether the effect of course modality on latent baseline student motivation was moderated by student characteristics. To assess this possibility, we included interactions of course modality and student characteristics (gender, underrepresented racial/ethnic minority status, generation status, and adult learner status) predicting latent motivation scores. As shown in Figure 2, there were no significant interactions of course modality and student characteristics predicting latent baseline expectancy, value, cost, or interest. However, the interaction of course modality and generation status were marginally negatively associated with latent incoming growth mindset and social belonging such that first-generation students enrolled in online courses tended to report less growth mindset and less social belonging at their institution. These results suggest that course modality is unrelated to latent Time 1 student motivation (with the marginal exception of interest), and that generally, student gender, underrepresented racial/ethnic minority status, and adult student status do not moderate the relationship between course modality and latent student motivations, however, course modality and generation status are marginally negatively associated with growth mindset and social belonging.

### Predicting Change in Motivation Over Time by Course Modality

Next, we examined whether course modality predicted change in motivation over the course of the semester (i.e., whether students in online courses reported greater, lesser, or equal changes in motivation across the semester as students in face-to-face courses). We conducted these analyses for expectancy, value, cost, interest, and relevance because these variables were assessed at multiple points throughout the semester. In analyses considering change in motivation over time, we operationalized change as the difference in motivation from Time 1 (Week 1) to Time 3 (Week 5). Descriptive statistics for motivation composites across Time 1–Time 3 by course modality can be seen in Table 2. In order to determine whether course modality predicted change in motivation over time, we fit linear multilevel models in which Time 3 motivation was regressed on course modality for each motivational construct (i.e., expectancy, value, cost, interest, relevance). All models controlled for course type, semester, student gender, student underrepresented ethnic/racial minority status, student generation status, and student high school GPA as well as the Time 1 motivation composite for the motivation being predicted. As shown in Table 3, being enrolled in an online course was not a significant predictor of change in motivation over the semester after employing a Bonferroni correction (α = 0.01). Findings suggest that changes in motivation over the course of the semester were not a function of course modality.

**TABLE 2:** Motivation across time points by course modality.

	**Time 1 Mean (*SD*)**		**Time 2 Mean (*SD*)**		**Time 3 Mean (*SD*)**	
**Baseline**						
**motivation**	**Face-to-face**	**Online**	**Face-to-face**	**Online**	**Face-to-face**	**Online**
N	1,426	266	1,618	267	1,471	217
Expectancy	3.81 (0.81)	3.66 (0.85)	3.74 (0.94)	3.56 (1.01)	3.62 (0.99)	3.45 (0.97)
Value	3.58 (0.92)	3.59 (0.93)	3.68 (0.98)	3.71 (0.93)	3.51 (1.04)	3.69 (0.96)
Cost	2.50 (0.84)	2.66 (0.85)	2.58 (1.04)	2.80 (1.08)	2.64 (1.06)	2.88 (1.08)
Relevance	3.20 (1.16)	3.13 (1.19)	2.96 (1.24)	2.94 (1.17)	3.04 (1.22)	3.09 (1.26)
Interest	2.71 (1.19)	2.64 (1.20)	2.56 (1.24)	2.52 (1.22)	2.58 (1.22)	2.51 (1.26)

**TABLE 3:** Course modality and demographics predicting change in motivation (Weeks 1–5).

	**Change in**	**Change**	**Change**	**Change in**
	**expectancy**	**in value**	**in cost**	**interest**
Course modality	−0.03(0.08)	0.16(0.08)	0.12(0.09)	−0.03(0.08)
Gender	0.08(0.05)	0.03(0.05)	−0.11(0.05)	0.20^***^(0.05)
Ethnic/Racial minority status	−0.11(0.05)	0.07(0.05)	0.09(0.05)	0.02(0.05)
Generation status	0.04(0.05)	0.03(0.05)	0.04(0.05)	−0.02(0.05)
Adult learner status	−0.02(0.07)	0.06(0.07)	0.02(0.08)	−0.03(0.07)
Course modality × Gender	0.01(0.15)	−0.07(0.15)	−0.01(0.17)	0.04(0.15)
Course modality × Ethnic/racial minority status	−0.12(0.13)	−0.19(0.13)	0.06(0.15)	−0.09(0.13)
Course modality × Generation status	0.11(0.13)	0.26(0.13)	0.08(0.15)	0.07(0.13)
Course modality × Adult learner status	0.02(0.16)	−0.01(0.17)	−0.50^∗^(0.19)	0.24(0.17)
N	1,129	1,128	1,127	1,118

**All models controlled for course type, semester, high school GPA (centered), and baseline (Time 1) motivation. The reference groups for course modality, gender, ethnic/racial minority status, generation status, and adult learner are “face-to-face,” “female,” “Non-URM,” “continuing-generation,” and “traditional age,” respectively.*^∗∗∗^*p* < 0.001 and ^∗^*p* < 0.05.*

We further investigated whether student demographics interacting with course modality predicted change in motivation over the course of the semester (i.e., whether students in online courses reported greater, lesser, or equal changes in motivation based on demographic characteristics compared to students in face-to-face courses. In order to do this, we fit linear models in which Time 3 motivation was regressed on the interaction of course modality and student demographic characteristic (gender, underrepresented ethnic/racial minority status, generation status, and adult learner status). All models controlled for course type, semester, student gender, student underrepresented ethnic/racial minority status, student generation status, and student high school GPA as well as the Time 1 motivation composite for the motivation being predicted. As shown in Table 3, there are no significant interactions of course modality and student demographic predicting change in motivation after employing Bonferroni corrections (α = 0.013) with the exception of the interaction of course modality and adult learner status on change in cost [β = −0.50, *t*(1,125) = −2.71, *p* = 0.007]. Adult learners in online courses tended to experience less of an increase in cost over the course of the semester than adult learners in face-to-face courses and traditional-aged learners in general. These results indicate that changes in motivation over the course of the semester were not a function of course modality and student demographics with the exception of adult learners in online courses and their experience of cost over time.

## Discussion

Online courses have become increasingly available and popular in higher education, particularly in community college (Bennett et al., 2010; Allen and Seaman, 2016). While some have lauded online courses as an opportunity to increase access for non-traditional and historically underrepresented learners (Yoo and Huang, 2013; Drew et al., 2015), others cite poor performance and high dropout rates as significant drawbacks (Patterson and McFadden, 2009; Boston and Ice, 2011). Evidence is inconsistent on whether students who engage in online learning are as motivated and successful as students who engage in traditional face-to-face learning, and for whom online courses may be most beneficial or detrimental (Phipps and Merisotis, 1999; Bernard et al., 2004; Johnson and Mejia, 2014). This study documented the motivational experiences of students in online and face-to-face courses, along with their academic performance. We focused on developmental mathematics courses, which serve academically underprepared students and are notorious barriers to graduation. Taken together, our findings suggest that there are few differences in online and face-to-face students’ incoming motivation and motivational change over time, and that motivational experiences do not differ systematically based on students’ gender, generation status, underrepresented ethnic/racial minority status, or age.

### Do Face-to-Face Students Outperform Online Students?

One of the primary arguments against online learning is that online students perform worse and drop out at higher rates than face-to-face students (Harris and Parrish, 2006). However, multiple syntheses concluded that there are no significant differences between the two modalities (e.g., Russell and Russell, 1999; Bernard et al., 2004; Zhao et al., 2005) and that negative effects are only present for certain subgroups of students. Findings from the current study indicated that online learners received lower course grades, lower pass rates, and higher withdrawal rates than their classmates in face-to-face courses. Although significant, it is important to note that the size of effects was small (Cohen’s *d*s = 0.17–0.28). When interpreting these findings, we are also mindful of Johnson and Mejia’s (2014) work with community college students in California, who concluded that online students displayed negative short-term effects (i.e., course-level performance and persistence) but positive long-term outcomes (i.e., degree attainment, enrollment in 4 years institution). Future analyses with this sample will assess participants’ longer-term outcomes, such as how many math courses they pursue and whether they are successful in future higher education or employment contexts.

When considering findings by subgroup, results suggested that the only significant interaction between student characteristic and course modality was for adult learner status. Online adult learners received significantly lower course grades and pass rates than face-to-face adult learners or traditional-aged learners in either online or face-to-face courses, with a consistent marginal finding for withdraw rates. This finding is of interest because adult learners are one of the most commonly cited reasons for providing online education options and comprise a sizable percentage of the online learner population (Yoo and Huang, 2013).

### Do Online Students Report Lower Motivation Than Face-to-Face Students?

Motivation is a critical predictor of academic success (Wigfield and Cambria, 2010), and has been identified as a theoretically-meaningful component of online learners’ success (Hartley and Bendixen, 2001; Hu and Kuh, 2002; Keller and Suzuki, 2004). We documented students’ motivation using an expectancy-value-cost framework (Wigfield and Eccles, 2000; Barron and Hulleman, 2015) and additional key constructs such as growth mindset and belonging. Results indicated that incoming students reported comparable expectations that they could be successful in the course, value for the course, and perceived cost of being involved in the course regardless of whether they enrolled in an online or face-to-face version of the class. This lack of difference counters any hypothesis that students may be differentially selecting to enroll in online courses because they are more or less motivated to take the course, at least among the current sample.

We were also interested in how students’ motivation changed over time, and documented students’ motivation at the beginning (Week 1) and middle (Weeks 3, 5) of the semester. We focused on this time period because it aligned with the add/drop period for the course, and consequently could be an important predictor of course drop out. Similar to findings for incoming motivational levels, descriptive results indicated that face-to-face and online students did not show differential patterns of motivational change. This suggests that, at least for the first half of the semester, online students’ changes in expectancy, value, and cost are not meaningfully different from those of face-to-face students. However, we were not able to meaningfully assess motivational change from the beginning to end of the semester given a low response rate (45%) to our survey administered in Week 12. Future research may wish to collect data on longer-term changes in motivation. Future studies could also assess motivational change at a more fine-grained level by collecting data more frequently to determine when – if ever – online and face-to-face students’ motivational trajectories diverge.

We were also interested in whether incoming academic motivation could account for differences in online and face-to-face students’ academic outcomes. Findings from our structural equation model (Figure 1) indicated no significant interaction between any of the incoming motivational variables and online versus face-to-face courses. The fact that this finding applied across a sample of students drawn from six semesters provides some assurance that these findings are replicable in the current sample of community college developmental mathematics students. Future research, however, could help determine whether this lack of relation replicates in other learning contexts, which would suggest that academic motivation is not a meaningful explanatory factor accounting for differences between online and face-to-face students’ academic success, or is unique to the community college or developmental mathematics setting.

### Limitations and Future Directions

The current study provides a broad description of online students’ motivational experiences in an important setting in higher education. It also provides preliminary evidence suggesting that the small but significant differences in academic performance between online and face-to-face courses does not appear to be a function of students’ incoming motivational beliefs. Although this information contributes to our understanding of online students’ affective experiences, there are a number of additional potential explanatory mechanisms that were not assessed. Future research may wish to consider constructs such as self-regulated learning strategies (e.g., time management; Broadbent and Poon, 2015) as reasons why online and face-to-face learners may receive different academic outcomes. Students’ prior experiences in online courses may also be an important factor to consider. Like motivation, the extant literature on prior experience is mixed, with some studies finding no relation between prior online experience and course performance (e.g., Arbaugh, 2005) and others finding effects of prior online experience on retention and completion rates (e.g., Dupin-Bryant, 2004). Similarly, students’ reasons for enrolling in online courses and the percentage of courses that students take online versus face-to-face may also affect students’ academic outcomes and course motivation. Future studies should assess these background variables and account for them in subsequent analyses.

The current study was also limited to assessing short-term motivation (i.e., from the beginning to middle of the semester) and outcomes (i.e., course grade, pass rate, and withdraw rate). However, prior research suggests that there are benefits to measuring longer-term change in motivation (Kosovich et al., 2017) and that the pattern of effects of taking online courses for short-term and long-term outcomes can vary substantially (Johnson and Mejia, 2014). Future research may wish to collect longer-term data from online and face-to-face students in terms of their motivation, perceptions of instructors, and academic outcomes. Finally, the current study was correlational. Because students chose to enroll in online or face-to-face versions of their courses, we cannot make claims regarding causal effects of online course enrollment on academic outcomes or motivational change. To enable such claims, future studies may wish to randomly assign students to complete online or face-to-face versions of courses, then assess their academic outcomes. Causal evidence from a randomized controlled trial could augment the current evidentiary basis by providing more definitive evidence on the effect of online course enrollment for student motivation and success.

## Data Availability

The datasets generated for this study are available on request to the corresponding author.

## Ethics Statement

The studies involving human participants were reviewed and approved by University of Virginia Institutional Review Board Valencia College Institutional Review Board. The patients/participants provided their written informed consent to participate in this study.

## Author Contributions

MF contributed to the conceptual framing of the study, helped determine and execute the analytic plan, helped conduct a literature review, and was the primary author of the methods and results sections. SW contributed to the conceptual framing of the study, provided the theoretical framework from which the study was based, helped conduct a literature review, helped determine an analytic plan, was the primary author of the introduction and discussion sections, and provided substantive direction and feedback on the methods and results sections. CH contributed to the conceptual framing of the current study and the larger project from which it was drawn, oversaw all efforts to collect data, and provided substantive feedback on the manuscript.

## Conflict of Interest Statement

The authors declare that the research was conducted in the absence of any commercial or financial relationships that could be construed as a potential conflict of interest.
