# Supplementary material for: The Costs of Online Learning: Examining Differences in Motivation and Academic Outcomes in Online and Face-to-Face Community College Developmental Mathematics Courses
Source: Front Psychol. 2019 Sep 10;10:2054. doi: 10.3389/fpsyg.2019.02054 (PMC6746985; doi:10.3389/fpsyg.2019.02054)
Supplement: Supplementary file 1 [file Table_1.docx]

Supplemental Table 1

*Variables of Interest by Course Modality and Gender*

|  | **Face-to-Face** | | **Online** | | | **ANOVA / Logistic Regression for Gender** | **Effect Size of Difference** |
| --- | --- | --- | --- | --- | --- | --- | --- |
|  | Female | Male | Female | | Male |  |  |
|  | Mean (SD) | | | | |  |  |
| **Academic Outcomes** | | | | | | | |
| Pass Rate | 0.69 (0.46) | 0.61 (0.48) | 0.59 (0.49) | 0.45  (0.50) | | *β* = -0.51,  *z* = -4.41, *p <* .001 | *β_OR_* = 0.672 |
| Grade | 2.18 (1.42) | 1.91 (1.51) | 1.83 (1.52) | 1.36 (1.48) | | *F*(1,2378) = 23.68, *p <*.001 | *η^2^* = 0.010 |
| Withdraw Rate | 0.11 (0.31) | 0.15 (0.36) | 0.14 (0.35) | 0.26 (0.44) | | *β* = 0.43,  *z* = -4.41, *p <* .001 | *β_OR_* = 1.569 |
| **Motivational Constructs** | | | | | | | |
| Baseline Expectancy | 3.75 (0.82) | 3.91 (0.78) | 3.56 (0.87) | 3.91 (0.76) | | *F*(1,1671) = 20.72, *p <*.001 | *η^2^* = 0.012 |
| Baseline Value | 3.58 (0.92) | 3.59 (0.94) | 3.56 (0.92) | 3.67 (0.95) | | *F*(1,1671) = 0.28, *p* = .598 | *η^2^* = 0.000 |
| Baseline Cost | 2.54 (0.84) | 2.43 (0.81) | 2.68 (0.86) | 2.56 (0.80) | | *F*(1,1668) = 7.41, *p* = .007 | *η^2^* = 0.004 |
| Baseline Relevance | 3.14 (1.15) | 3.30 (1.17) | 3.02 (1.16) | 3.38 (1.22) | | *F*(1,1671) = 9.77, *p* = .002 | *η^2^* = 0.006 |
| Baseline Interest | 2.64 (1.18) | 2.81 (1.19) | 2.51 (1.22) | 2.93 (1.10) | | *F*(1,1671) = 11.30, *p <* .001 | *η^2^* = 0.007 |
| Baseline Growth Mindset | 3.80 (1.20) | 4.01 (1.16) | 3.71 (1.35) | 4.07 (1.16) | | *F*(1,1660) = 14.02, *p <* .001 | *η^2^* = 0.008 |
| Baseline Belonging | 3.67 (0.79) | 3.67 (0.75) | 3.57 (0.80) | 3.59 (0.82) | | *F*(1,1669) = 0.01, *p* = .945 | *η^2^* = 0.000 |
