# Supplementary material for: The Costs of Online Learning: Examining Differences in Motivation and Academic Outcomes in Online and Face-to-Face Community College Developmental Mathematics Courses
Source: Front Psychol. 2019 Sep 10;10:2054. doi: 10.3389/fpsyg.2019.02054 (PMC6746985; doi:10.3389/fpsyg.2019.02054)
Supplement: Supplementary file 2 [file Table_2.docx]

Supplemental Table 2

*Variables of Interest by Course Modality and Underrepresented Minority (URM) Status*

|  | **Face-to-Face** | | **Online** | | | **ANOVA / Logistic Regression for URM Status** | **Effect Size of Difference** |  |
| --- | --- | --- | --- | --- | --- | --- | --- | --- |
|  | Maj. | URM | Maj. | | URM |  |  |  |
|  | Mean (SD) | | | | |  |  |  |
| **Academic Outcomes** | | | | | | | | |
| Pass Rate | 0.70 (0.46) | 0.64 (0.48) | 0.55 (0.50) | 0.51  (0.50) | | *β* = -0.26,  *z* = 0.05, *p* = .996 | *β_OR_* = 0.775 |  |
| Grade | 2.25 (1.49) | 1.97 (1.43) | 1.76 (1.61) | 1.48 (1.41) | | *F*(1,2222) = 18.34, *p <*.001 | *η^2^* = 0.008 |  |
| Withdraw Rate | 0.13 (0.33) | 0.13 (0.33) | 0.18 (0.39) | 0.19 (0.40) | | *β* = 0.20,  *z* = 0.62, *p* = .533 | *β_OR_* = 1.044 |  |
| **Motivational Constructs** | | | | | | | | |
| Baseline Expectancy | 3.77 (0.78) | 3.86 (0.81) | 3.65 (0.85) | 3.69 (0.85) | | *F*(1,1554) = 3.31, *p* = .069 | *η^2^* = 0.002 |  |
| Baseline Value | 3.43 (0.93) | 3.70 (0.90) | 3.45 (0.92) | 3.78 (0.87) | | *F*(1,1552) = 33.21, *p <* .001 | *η^2^* = 0.021 |  |
| Baseline Cost | 2.41 (0.83) | 2.54 (0.83) | 2.64 (0.86) | 2.67 (0.88) | | *F*(1,1551) = 6.76, *p* = .009 | *η^2^* = 0.004 |  |
| Baseline Relevance | 2.99 (1.15) | 3.35 (1.14) | 2.93 (1.15) | 3.37 (1.17) | | *F*(1,1554) = 38.79, *p <* .001 | *η^2^* = 0.024 |  |
| Baseline Interest | 2.51 (1.18) | 2.83 (1.18) | 2.44 (1.13) | 2.89 (1.25) | | *F*(1,1554) = 29.98, *p <* .001 | *η^2^* = 0.019 |  |
| Baseline Growth Mindset | 3.82 (1.24) | 3.91 (1.17) | 3.62 (1.31) | 3.92 (1.24) | | *F*(1,1543) = 4.04, *p* = .044 | *η^2^* = 0.003 |  |
| Baseline Belonging | 3.57 (0.75) | 3.74 (0.76) | 3.55 (0.83) | 3.65 (0.75) | | *F*(1,1552) = 15.66, *p <* .001 | *η^2^* = 0.010 |  |
