# Supplementary material for: The Costs of Online Learning: Examining Differences in Motivation and Academic Outcomes in Online and Face-to-Face Community College Developmental Mathematics Courses
Source: Front Psychol. 2019 Sep 10;10:2054. doi: 10.3389/fpsyg.2019.02054 (PMC6746985; doi:10.3389/fpsyg.2019.02054)
Supplement: Supplementary file 3 [file Table_3.docx]

Supplemental Table 3

*Variables of Interest by Course Modality and Generation Status*

|  | **Face-to-Face** | | **Online** | | | **ANOVA / Logistic Regression for Gen Status** | **Effect Size of Difference** |
| --- | --- | --- | --- | --- | --- | --- | --- |
|  | CG | FG | CG | | FG |  |  |
|  | Mean (SD) | | | | |  |  |
| **Academic Outcomes** | | | | | | | |
| Pass Rate | 0.69 (0.46) | 0.66 (0.48) | 0.57 (0.50) | 0.55  (0.50) | | *β* = -0.13,  *z* = -1.35, *p* = .177 | *β_OR_* = 0.881 |
| Grade | 2.18 (1.45) | 2.03 (1.46) | 1.75 (1.54) | 1.63 (1.49) | | *F*(1,2155) = 5.64, *p* = .018 | *η^2^* = 0.003 |
| Withdraw Rate | 0.10 (0.30) | 0.13 (0.34) | 0.17 (0.38) | 0.19 (0.39) | | *β* = 0.29,  *z* = 2.18, *p* = .029 | *β_OR_* = 1.335 |
| **Motivational Constructs** | | | | | | | |
| Baseline Expectancy | 3.83 (0.83) | 3.79 (0.82) | 3.66 (0.85) | 3.64 (0.86) | | *F*(1,1609) = 0.75, *p* = .385 | *η^2^* = 0.000 |
| Baseline Value | 3.55 (0.94) | 3.64 (0.90) | 3.62 (0.89) | 3.58 (0.96) | | *F*(1,1608) = 2.60, *p* = .107 | *η^2^* = 0.002 |
| Baseline Cost | 2.49 (0.84) | 2.54 (0.83) | 2.65 (0.86) | 2.67 (0.85) | | *F*(1,1607) = 1.30, *p* = .255 | *η^2^* = 0.001 |
| Baseline Relevance | 3.19 (1.18) | 3.21 (1.15) | 3.14 (1.17 | 3.14 (1.25) | | *F*(1,1608) = 0.06, *p* = .800 | *η^2^* = 0.000 |
| Baseline Interest | 2.67 (1.18) | 2.74 (1.20) | 2.68 (1.18) | 2.61 (1.23) | | *F*(1,1609) = 0.64, *p* = .425 | *η^2^* = 0.000 |
| Baseline Growth Mindset | 3.92 (1.14) | 3.87 (1.24) | 3.77 (1.34) | 3.83 (1.28) | | *F*(1,1601) = 0.26, *p* = .612 | *η^2^* = 0.000 |
| Baseline Belonging | 3.67 (0.77) | 3.67 (0.78) | 3.46 (0.80) | 3.71 (0.78) | | *F*(1,1608) = 1.15, *p* = .283 | *η^2^* = 0.001 |
