# Supplementary material for: The Costs of Online Learning: Examining Differences in Motivation and Academic Outcomes in Online and Face-to-Face Community College Developmental Mathematics Courses
Source: Front Psychol. 2019 Sep 10;10:2054. doi: 10.3389/fpsyg.2019.02054 (PMC6746985; doi:10.3389/fpsyg.2019.02054)
Supplement: Supplementary file 4 [file Table_4.docx]

Supplemental Table 4

|  | **Face-to-Face** | | **Online** | | | **ANOVA / Logistic Regression for Adult Status** | **Effect Size of Difference** |
| --- | --- | --- | --- | --- | --- | --- | --- |
|  | Trad. | Adult | Trad. | | Adult |  |  |
|  | Mean (SD) | | | | |  |  |
| **Academic Outcomes** | | | | | | | |
| Pass Rate | 0.65 (0.48) | 0.75 (0.44) | 0.55 (0.50) | 0.51  (0.50) | | *β* = 0.23,  *z* = 1.73, *p* = .08 | *β_OR_* = 1.263 |
| Grade | 2.02 (1.45) | 2.48 (1.49) | 1.68 (1.50) | 1.56 (1.57) | | *F*(1,2405) = 11.43, *p <* .001 | *η^2^* = 0.005 |
| Withdraw Rate | 0.10 (0.30) | 0.13 (0.34) | 0.17 (0.38) | 0.19 (0.39) | | *β* = 0.16,  *z* = 0.92, *p* = .359 | *β_OR_* = 1.171 |
| **Motivational Constructs** | | | | | | | |
| Baseline Expectancy | 3.83 (0.80) | 3.67 (0.88) | 3.70 (0.78) | 3.54 (1.01) | | *F*(1,1689) = 7.01, *p* = .008 | *η^2^* = 0.004 |
| Baseline Value | 3.56 (0.91) | 3.58 (1.04) | 3.54 (0.91) | 3.74 (0.99) | | *F*(1,1687) = 0.61, *p* = .435 | *η^2^* = 0.000 |
| Baseline Cost | 2.48 (0.84) | 2.71 (0.77) | 2.65 (0.80) | 2.70 (0.96) | | *F*(1,1686) = 9.06, *p* = .003 | *η^2^* = 0.005 |
| Baseline Relevance | 3.18 (1.15) | 3.29 (1.25) | 3.02 (1.17) | 3.43 (1.20) | | *F*(1,1689) = 4.99, *p* = .026 | *η^2^* = 0.003 |
| Baseline Interest | 2.71 (1.18) | 2.70 (1.24) | 2.54 (1.14) | 2.89 (1.33) | | *F*(1,1689) = 1.08, *p* = .300 | *η^2^* = 0.001 |
| Baseline Growth Mindset | 3.85 (1.19) | 4.09 (1.21) | 3.79 (1.27) | 3.88 (1.39) | | *F*(1,1678) = 5.59, *p* = .018 | *η^2^* = 0.003 |
| Baseline Belonging | 3.68 (0.76) | 3.57 (0.85) | 3.58 (0.77) | 3.59 (0.89) | | *F*(1,1687) = 1.99, *p* = .159 | *η^2^* = 0.002 |

*Variables of Interest by Course Modality and Adult Learner Status*
